# Sex-Based Differences in Hemodynamic Response to Anesthesia Type During TAVI and Early Transvalvular Gradient Changes

**DOI:** 10.3390/jcm14196693

**Published:** 2025-09-23

**Authors:** Benjamin Fogelson, Raj Baljepally, Phoebe Tran, Eric Heidel, Terrance C. Nowell, Billy Morvant, Steve Ferlita, Stefan Weston, Aladen Amro, Kirsten Ferraro, Zachary Spires, Soham Nadkarni

**Affiliations:** 1 Department of Medicine, Division of Cardiology, University of Tennessee Graduate School of Medicine, Knoxville, TN 37920, USA; 2 Department of Public Health, University of Tennessee, Knoxville, TN 37920, USA; 3 Department of Surgery, University of Tennessee Graduate School of Medicine, Knoxville, TN 37920, USA; 4 Department of Anesthesia, University of Tennessee Graduate School of Medicine, Knoxville, TN 37920, USA; 5 Department of Medicine, University of Tennessee Graduate School of Medicine, Knoxville, TN 37920, USA

**Keywords:** transcatheter aortic valve implantation, transvalvular gradient, anesthesia, general anesthesia, monitored anesthesia care

## Abstract

**Background and Objectives:** Anesthesia type may influence early hemodynamics post-transcatheter aortic valve implantation (TAVI), but sex-based differences in anesthetic response remain underexamined. We aimed to assess whether male and female patients exhibit differential responses to general anesthesia (GA) versus monitored anesthesia care (MAC) during TAVI, with particular attention to post-procedural transvalvular gradient changes. **Methods:** We conducted a single-center retrospective cohort study of 693 patients who underwent TAVI between 2011 and 2023 with complete echocardiographic and anesthesia data. Patients were categorized into four groups by sex and anesthesia type: GA-Male, MAC-Male, GA-Female, and MAC-Female. Hemodynamic, anesthetic, echocardiographic characteristics, and 6-month outcomes were compared. **Results:** Significant differences were observed across the four sex-anesthesia groups in several hemodynamic and echocardiographic measures. Initial analyses showed that female patients had significantly higher 24 h post-TAVI transvalvular mean gradient delta values compared to males, and among MAC patients, females also had higher 30-day mean gradients. However, secondary analyses revealed that valve size differed significantly between groups and was a key driver of these hemodynamic differences. After adjusting for valve size in a multivariable regression model, gradient differences between groups were no longer statistically significant. Net fluid balance and vasopressor use were more strongly associated with anesthesia type than sex, with GA groups requiring greater support. No significant differences in 6-month cardiovascular outcomes were observed. **Conclusions:** Early post-TAVI transvalvular gradient changes appeared to be primarily influenced by valve size rather than sex or anesthesia type alone. These findings suggest previously observed sex-based differences may reflect underlying disparities in valve sizing, highlighting need for further prospective studies assessing the independent contributions of sex, anesthesia modality, and valve size on early valve performance/long-term outcomes.

## 1. Introduction

Transcatheter aortic valve implantation (TAVI) has become the standard of care for severe aortic stenosis in high- and intermediate-risk populations [1,2]. Around 78,000 TAVI surgeries are performed in the US annually [1,2]. The number of TAVI surgeries is expected to grow by 4–10% each year due to the continued aging of the US population [1,2].

An increase in transvalvular mean gradient (TVMG) from immediate post-TAVI to 24 h post-TAVI is a well-documented phenomenon, often attributed to peri-procedural factors such as anesthesia, fluid shifts, and low-flow states [3,4]. While prior studies, including those conducted by our team, have shown that the choice of general anesthesia (GA) versus monitored anesthesia care (MAC) does not significantly influence TVMG rise overall, no studies have examined whether the TVMG response to anesthesia type differs by sex [5]. Existing research examining sex-specific differences in response to anesthesia type among adults in the US and in other countries has been conducted in a non-TAVI population and does not consider TVMG or changes in TVMG over 24 h or 30 days post-TAVI [6,7,8,9,10,11,12,13,14]. Consequently, there may be limited generalizability of these findings to US patients who have undergone TAVI, as they are on average 80 years old, with evidence indicating that around half have one or more underlying comorbidities [15,16].

Given known physiologic and cardiovascular differences between sexes, this study aims to evaluate whether male and female patients respond differently to anesthesia types in the context of TAVI. Specifically, we examine differences in hemodynamics and echocardiographic characteristics immediately after TAVI, 24 h after TAVI, and at 30 days after TAVI by sex and anesthesia type. Additionally, differences in baseline demographic and clinical characteristics and six-month post-TAVI outcomes by sex and anesthesia type are also presented.

## 2. Materials and Methods

### 2.1. Study Population

We conducted a retrospective observational cohort study using data from the University of Tennessee Medical Center institutional TAVI registry for the period from 1 January 2011 to 31 December 2023. The study was approved by our institutional review board (IRB Registration number 00005012, approved 1 March 2023, extended on 31 December 2023). Informed consent was waived by our institution’s IRB, given the retrospective nature of this study. The study was conducted in compliance with the ethical standards of our institution as well as the revised Helsinki Declaration. Individuals eligible for inclusion were those who underwent TAVI; had available echocardiograms immediately after TAVI, at 24 h, and at 30 days after TAVI; and had documentation of anesthesia type (MAC or GA). All patients in this study received balloon-expandable Edwards SAPIEN valves (SAPIEN, SAPIEN XT, or SAPIEN 3), with the majority receiving SAPIEN 3 prostheses. No patients receiving self-expanding valves were included in the cohort. Individuals were excluded if they had concomitant valve procedures, valve-in-valve TAVI, missing or incomplete gradient data, or if they crossed over from MAC to GA.

### 2.2. Study Variables

All data on study variables were obtained from the institutional TAVI registry. Our exposures of interest were sex (male or female) and anesthesia type (GA or MAC). Patients were divided into four groups based on sex and anesthesia type: (1) GA-Male, (2) GA-Female, (3) MAC-Male, and (4) MAC-Female. Baseline demographic and clinical characteristics included in the study were age (years), diabetes mellitus, hypertension, hyperlipidemia, peripheral vascular disease, stroke/transient ischemic attack (TIA), chronic obstructive pulmonary disease (COPD), atrial fibrillation, previous pacemaker, chronic kidney disease (CKD) (any stage), CKD stage 4 or end stage renal disease (ESRD), glomerular filtration rate (GFR), coronary artery disease, previous myocardial infarction, previous coronary artery bypass grafting (CABG), obstructive sleep apnea, body mass index (BMI) (kg/m^2^), New York Heart Association (NYHA) class I–IV symptoms, and CHA2DS2-VASc score.

Hemodynamic and anesthesia characteristics included systolic blood pressure (SBP) pre-TAVI (mmHg), diastolic blood pressure (DBP) pre-TAVI (mmHg), SBP immediately post-TAVI (mmHg), DBP immediately post-TAVI (mmHG), SBP 24 h post-TAVI (mmHg), DBP 24 h post-TAVI (mmHg), total fentanyl dose (mcg), total versed dose (mg), propofol bolus (mg), propofol infusion (mg), total propofol used (mg), dexmedetomidine bolus (mcg), dexmedetomidine infusion (mcg), total dexmedetomidine used (mcg), use of inhaled anesthetic, need for vasopressors, intravenous (IV) fluids during TAVI (ml), 24 h fluid balance, pre-TAVI weight, and post-TAVI weight. Included echocardiographic measures were pre-TAVI echocardiogram (ECHO) mean gradient (mmHg), pre-TAVI ECHO aortic valve area (AVA) (cm^2^), pre-TAVI ejection fraction (EF) (%), pre-TAVI aortic regurgitation (AR) severity, post-TAVI EF, post-TAVI AVA, immediate TAVI mean gradient, 24 h TAVI mean gradient, mean gradient delta, immediate TAVI peak gradient, 24 h TAVI peak gradient, maximum gradient delta, 30-day TAVI mean gradient, 30-day TAVI peak gradient, 30-day EF, presence of paravalvular leak, severity of paravalvular leak (trace to mild), and severity of paravalvular leak (moderate to severe). All transvalvular gradients, including pre-TAVI, immediate post-TAVI, 24 h post-TAVI, and 30-day post-TAVI, were obtained using transthoracic echocardiography with Doppler assessment. The ‘immediate post-TAVI’ measurement refers to the first post-procedural echocardiogram performed in the recovery unit or ICU, and not to intra-procedural invasive assessment. No intra-procedural catheter-based gradients were included. We also assessed post-TAVI length of stay (days) and six-month post-TAVI outcomes, including myocardial infarction (MI) post-TAVI at six months, stroke/TIA post-TAVI at six months, all-cause death post-TAVI at six months, and cardiovascular-related death post-TAVI at six months.

### 2.3. Statistical Analysis

The four sex and anesthesia type groups were compared on non-normal continuous and ordinal-level characteristics and outcomes using the Kruskal–Wallis test. Medians (Mdn) and interquartile ranges (IQR) are presented for each of the four groups. If the overall Kruskal–Wallis test indicated statistical significance at an alpha level of 0.05, further post hoc pairwise comparisons were conducted to determine whether a particular characteristic or outcome differed significantly between the following pairs: MAC-Male and GA-Male, MAC-Male and MAC-Female, MAC-Male and GA-Female, GA-Male and MAC-Female, GA-Male and GA-Female, and MAC-Female and GA-Female. To reduce the risk of Type I error resulting from multiple pairwise comparisons, Bonferroni’s correction was applied during these post hoc tests. Categorical characteristics and outcomes were compared between the four groups using Chi-square tests or Fisher’s Exact tests, as appropriate based on sample size. Frequencies and percentages were presented and interpreted for categorical comparisons.

As part of secondary analyses, we examined the influence of valve size (20 mm, 23 mm, 26 mm, 29 mm) on differences in mean gradient delta between the four sex and anesthesia type groups using descriptive analyses and regression. Descriptive analyses included a Kruskal–Wallis test with post hoc tests corrected for type I error to assess mean gradient delta by valve size (treated as a categorical variable). A Kruskal–Wallis test with post hoc tests was also used to examine differences in valve size (treated as an ordinal variable) across the four sex and anesthesia type groups. Following these descriptive analyses, we created a negative binomial model that included valve size and other anesthesia- and sex-related covariates (COPD, CKD (any stage), CKD stage 4 or ESRD, severity of paravalvular leak (moderate severe), age, BMI, GFR, CHA2DS2-VASc Score, total fentanyl dose, total versed dose, pre-TAVI ECHO mean gradient, pre-TAVI EF, pre-TAVI AR severity). All statistical analyses were conducted using SPSS Version 29 (Armonk, NY, USA: IBM Corp.) with a two-sided alpha level of 0.05.

## 3. Results

Our study included 693 individuals, with *n* = 113 participants in the GA-Male group, *n* = 280 in the MAC-Male group, *n* = 78 in the GA-Female group, and *n* = 222 in the MAC-Female group (Table 1). Age was similar across the four groups, with the median age being 77 years (IQR: 71–83) for GA-Male, 76 years (IQR: 71–82) for MAC-Male, 80 years (IQR: 72–86) for GA-Female, and 77 years (IQR: 71–83) for MAC-Female. Among the other baseline characteristics considered, peripheral vascular disease, stroke/TIA, COPD, CKD (any stage), CKD stage 4 or ESRD, coronary artery disease, previous myocardial infarction, previous CABG, and CHA2DS2-VASc score were significantly different (*p* < 0.05) between the four groups. Although most clinical characteristics representing underlying comorbidities (diabetes mellitus, hypertension, hyperlipidemia, atrial fibrillation, obstructive sleep apnea) were not significantly different between the groups, it is notable that the percentage of individuals with diabetes in each group was around 40% or higher, hypertension around 87% or higher, hyperlipidemia around 83% or higher, and atrial fibrillation around 27% or higher.

The distribution of hemodynamic, anesthesia, and echocardiographic characteristics by sex and anesthesia type is presented in Table 2. Numerous hemodynamic and anesthesia characteristics differed significantly (*p* < 0.05) between the four groups, including SBP pre-TAVI (mmHg), DBP pre-TAVI (mmHg), DBP immediate post-TAVI (mmHg), total fentanyl dose (mcg), total versed dose (mg), propofol bolus (mg), propofol infusion (mg), total propofol (mg), dexmedetomidine bolus (mcg), dexmedetomidine infusion (mcg), total dexmedetomidine used (mcg), use of inhaled anesthetic, need for vasopressors, IV fluids during TAVI (ml), 24 h fluid balance, pre-TAVI weight, and post-TAVI weight. Additionally, several echocardiographic measures differed significantly between the four groups, including pre-TAVI ECHO AVA (cm^2^), pre-TAVI EF (%), post-TAVI EF, post-TAVI AVA, immediate TAVI mean gradient, 24 h TAVI mean gradient, mean gradient delta, immediate TAVI peak gradient, 24 h TAVI peak gradient, max gradient delta, 30-day TAVI mean gradient, 30-day TAVI peak gradient, 30-day EF, presence of paravalvular leak, severity of paravalvular leak (trace to mild), and TAVI size. Post-TAVI length of stay differed significantly between the four groups; however, none of the six-month post-TAVI outcomes (Table 3) showed significant differences among the groups.

Pairwise comparisons of continuous and ordinal-level characteristics and outcomes are displayed in Table 4. While an individual discussion of each significant variable for every pairwise comparison would be impractical due to the large number of findings, readers can refer to the median values of each variable in Table 1 and Table 2, and the pairwise comparisons in Table 4, if they are interested in a specific significant post hoc finding. Below, we highlight several notable significant findings.

- MAC-Male and GA-Male were significantly different in terms of DBP pre-TAVI (mmHg), DBP immediate post-TAVI (mmHg), DBP 24 h post-TAVI (mmHg), total fentanyl dose (mcg), total versed dose (mg), propofol bolus (mg), propofol infusion (mg), total propofol (mg), dexmedetomidine bolus (mcg), dexmedetomidine infusion (mcg), total dexmedetomidine used (mcg), IV fluids during TAVI (mL), 24 h fluid balance, pre-TAVI EF (%), post-TAVI EF, 30-day EF, CHA2DS2-VASc Score, and Post-TAVI length of stay.
- Comparing MAC-Male and MAC-Female, we observed significant differences in DBP immediate post-TAVI (mmHg), DBP 24 h post-TAVI (mmHg), pre-TAVI weight, post-TAVI weight, pre-TAVR ECHO AVA (cm^2^), pre-TAVR EF (%), post-TAVI EF, post-TAVI AVA, immediate TAVI mean gradient, 24 h TAVI mean gradient, mean gradient delta, immediate TAVI peak gradient, 24 h TAVI peak gradient, max gradient delta, 30-day TAVI mean gradient, 30-day EF, NYHA class I–IV symptoms, CHA2DS2-VASc Score, and Post-TAVI length of stay.
- For MAC-Male and GA-Female, significant differences were observed in DBP pre-TAVI (mmHg), DBP immediate post-TAVI (mmHg), DBP 24 h post-TAVI (mmHg), total fentanyl dose (mcg), total versed dose (mg), propofol bolus (mg), propofol infusion (mg), total propofol (mg), dexmedetomidine bolus (mcg), dexmedetomidine infusion (mcg), total dexmedetomidine used (mcg), 24 h fluid balance, pre-TAVI weight, post-TAVI weight, 24 h TAVI mean gradient, 24 h TAVI peak gradient, NYHA class I–IV symptoms, CHA2DS2-VASc Score, and Post-TAVI length of stay.
- In the GA-Male and MAC-Female comparison, there were significant differences in SBP pre-TAVI (mmHg), DBP pre-TAVI (mmHg), DBP 24 h post-TAVI (mmHg), total fentanyl dose (mcg), total versed dose (mg), propofol bolus (mg), propofol infusion (mg), total propofol (mg), dexmedetomidine bolus (mcg), dexmedetomidine infusion (mcg), total dexmedetomidine used (mcg), IV fluids during TAVI (ml), 24 h fluid balance, pre-TAVI weight, post-TAVI weight, pre-TAVR EF (%), post-TAVI EF, post-TAVI AVA, immediate TAVI mean gradient, 24 h TAVI mean gradient, mean gradient delta, 24 h TAVI peak gradient, max gradient delta, 30-day TAVI mean gradient, 30-day TAVI peak gradient, 30-day EF, and Post-TAVI length of stay.
- The GA-Male group was significantly different from the GA-Female group in terms of pre-TAVI weight, post-TAVI weight, pre-TAVI EF (%), post-TAVI EF, 24 h TAVI mean gradient, mean gradient delta, 24 h TAVI peak gradient, max gradient delta, and CHA2DS2-VASc Score.
- Lastly, the MAC-Female group differed significantly from the GA-Female group in DBP pre-TAVI (mmHg), total fentanyl dose (mcg), total versed dose (mg), propofol bolus (mg), propofol infusion (mg), total propofol (mg), dexmedetomidine bolus (mcg), dexmedetomidine infusion (mcg), total dexmedetomidine used (mcg), pre-TAVI EF (%), post-TAVI EF, post-TAVI AVA, immediate TAVI mean gradient, 30-day TAVI peak gradient, 30-day EF, and Post-TAVI length of stay.

Significant comparisons on the net fluid balance variable were observed between MAC-Male and GA-Male, MAC-Male and GA-Female, and GA-Male and MAC-Female. In each of these comparisons, the median value for net fluid balance was higher in the GA group than in the MAC group. These findings, combined with the lack of significant differences in net fluid balance between MAC-Male and MAC-Female and between GA-Male and GA-Female, suggest that the observed differences in net fluid balance may be influenced more by anesthesia type than by sex.

**Figure 1:**
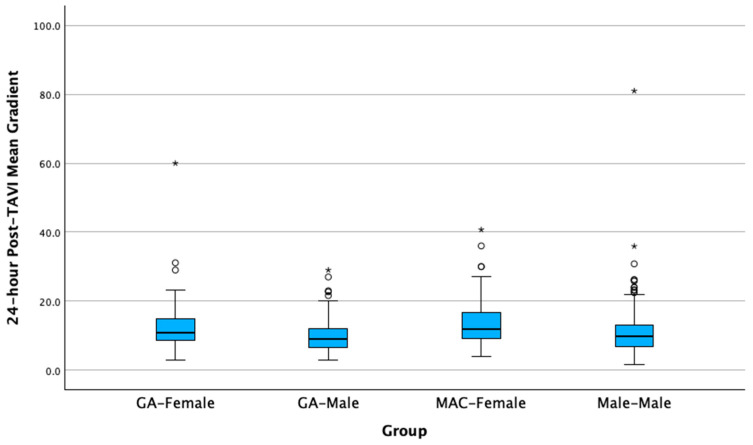
Differences in mean gradient delta by sex and anesthesia groups 24 h following transcatheter aortic valve implantation. Circles are outliers at 1.5× the interquartile range, asterisks are outliers at 3.0× the interquartile range.

Additionally, in the comparison between MAC-Male and MAC-Female for the variable mean gradient delta, the groups were significantly different, and the median value was higher for the MAC-Female group (Mdn = 7.1) versus the MAC-Male group (Mdn = 5.0) (Figure 1). Similarly, in the comparison between GA-Male and GA-Female for the same variable, the groups were significantly different, with the GA-Female group (Mdn = 6.4) having a higher median value compared to the GA-Male group (Mdn = 4.4). This gradient trend persists at 30 days for MAC, but not for GA. Specifically, in the MAC-Male and MAC-Female comparison for the 30-day TAVI mean gradient, the groups were significantly different, and the median value was again higher for the MAC-Female group (Mdn = 12.4) than for the MAC-Male group (Mdn = 10.0). In contrast, the GA-Male and GA-Female comparison for the 30-day TAVI mean gradient was not statistically significant.

In secondary analyses, we examined the influence of valve size on differences in mean gradient delta between the sex and anesthesia groups, with results presented in the Supplementary Materials. Among the four valve sizes used at our institution, statistically significant differences in mean gradient delta were observed between all groups except the 20 mm and 23 mm groups and the 26 mm and 29 mm groups (Table S1). When valve size was compared between the sex and anesthesia groups, significant differences were observed in all pairwise comparisons except between MAC Female and GA Female (Table S2). To adjust for the influence of valve size and other covariates on mean gradient delta, we fit a regression model that included valve size along with sex and anesthesia variables. Results indicated no differences between the sex and anesthesia groups in mean gradient delta after adjustment (Table S3).

## 4. Discussion

We conducted a retrospective cohort study to examine sex differences in anesthesia responses among patients who underwent TAVI at our institution between 2011 and 2023. Significant differences were observed across the four sex and anesthesia type groups in several hemodynamic, anesthetic, and echocardiographic measures. Notably, the mean gradient delta was significantly higher in females than in males, regardless of whether patients received MAC or GA. Similarly, the 30-day TAVI mean gradient was higher in females than in males among those who received MAC.

### 4.1. Interacting Effects of Sex, Anesthesia, and Valve Size on Post-TAVI Hemodynamics

Differences in net fluid balance and vasopressor requirements appeared to be more strongly associated with anesthesia type than sex, as GA groups had higher median values than MAC groups, independent of sex. Notably, in our prior work evaluating this same cohort, we found no significant difference in 24 h post-TAVI mean transvalvular gradients between MAC and GA overall [5]. However, when stratified by sex in the present analysis, meaningful differences emerged. These findings initially suggested that sex-specific physiologic responses may exert a greater influence on early post-TAVI valvular hemodynamics than the anesthesia modality alone.

Prior studies have demonstrated that women are more susceptible to anesthesia-induced hypotension, particularly under GA, possibly due to lower baseline vascular tone and heightened sensitivity to vasodilators [17,18]. Estrogen-related modulation of nitric oxide and prostacyclin pathways contributes to reduced systemic vascular resistance in women, which may exacerbate peri-induction hypotension [19,20]. These shifts in vascular tone and loading conditions may partly explain the more pronounced post-procedural increases in mean gradient observed in female patients 24 h after TAVI. Sex-based differences in anesthetic pharmacodynamics, such as faster emergence, slower recovery, and higher complication rates in women, may further influence post-TAVI hemodynamics [7,21]. These effects may be mediated through hormonal mechanisms and effects on GABA-A receptor function [7,22]. Prior studies have demonstrated that women require higher doses of morphine to achieve comparable analgesia following dexmedetomidine-based anesthesia [23]. More recently, preclinical research has highlighted intrinsic sex-related differences in anesthetic sensitivity [6]. Additional evidence points to sex-based variation in autonomic tone and anesthetic depth of awareness [10,11,12], further supporting the biological plausibility of our findings.

While prior literature suggests that women are more prone to anesthesia-induced hypotension, our cohort demonstrated normotensive pre-, post-, and 24 h post-TAVI blood pressures, suggesting that overt or sustained hypotension extending into the post-procedural period was not present. However, it is also important to note that continuous intra-procedural invasive hemodynamics were not available in this analysis. While significant or prolonged hypotension would likely have been detected during the procedure and treated by the anesthesia team, our dataset does not capture brief hypotensive episodes that may have been treated peri-procedurally or during induction. As previously shown in other studies, patients receiving GA required greater vasopressor support and had higher net fluid balances compared to those under MAC [5]. This reflects the need for increased hemodynamic support in GA patients under the physiologic conditions of inhaled anesthetics and mechanical ventilation. Although vasopressor use was more common in GA compared to MAC, there was no significant difference in vasopressor administration between men and women within each anesthesia group. However, because this analysis did not capture vasopressor dose or duration, nor episodes of hypotension throughout the procedure, we cannot fully characterize the extent of anesthesia-induced hemodynamic instability treated peri-procedurally.

In the present study, which incorporates both sex and anesthesia type, our additional analyses suggest that differences in post-TAVI outcomes across the four groups are largely driven by valve size. In secondary analyses, we observed statistically significant differences in mean gradient delta between valve size groups, and valve size also differed significantly across sex-anesthesia categories. When we adjusted for valve size (along with sex and anesthesia variables) in regression models, the differences in mean gradient delta between groups were no longer statistically significant. This suggests that valve size may be a primary driver of the observed hemodynamic differences, rather than anesthesia or sex independently. However, we also acknowledge that our study may be underpowered to detect independent associations in certain subgroups, particularly due to the absence of male patients receiving the smallest (20 mm) valves. Future studies with larger and more balanced samples, especially including more men treated with smaller valve sizes, are needed to clarify these relationships more definitively. In contextualizing our findings, it is also important to consider known hemodynamic differences between valve types. While all patients in this study received balloon-expandable valves (BEVs), it is important to note that prior literature has demonstrated significant hemodynamic differences between BEVs and self-expanding valves (SEVs). Specifically, SEVs have been consistently associated with lower transvalvular gradients and larger effective orifice areas compared to BEVs, particularly in patients with small annuli. These differences are largely attributed to valve design features such as supra-annular leaflet position and differing stent architecture. Thus, the relatively higher gradients observed in our cohort, comprised exclusively of BEV recipients, should be interpreted within this context. Our findings may not fully extrapolate to populations receiving SEVs, and further investigation is warranted to evaluate whether sex- and anesthesia-related hemodynamic differences persist across different prosthesis platforms [24]. Lastly, while female patients in the MAC group demonstrated higher mean gradients at 30 days compared to females who received GA, the clinical implications of this difference are uncertain. Given that anesthesia-related influences are most likely to impact early hemodynamics, the divergence at 30 days may reflect factors unrelated to anesthetic technique. Our updated analyses suggest that valve sizing may offer a more compelling explanation for these differences and should be considered in future evaluations.

### 4.2. Conduction Abnormalities After TAVI: Clinical Relevance and Implications for Sex and Anesthesia Differences

Although our dataset did not include post-procedural ECG data, conduction disturbances, particularly atrioventricular block (AVB) and left bundle branch block (LBBB), represent an important clinical consideration in the post-TAVI period. Prior studies have shown that new-onset LBBB occurs in up to 27% of patients following TAVI, and while often transient, it may progress to high-grade AVB requiring permanent pacemaker implantation in a subset of cases [25]. These conduction disturbances can influence hemodynamics by inducing interventricular dyssynchrony, reducing stroke volume, and altering pressure gradients across the valve.

Emerging data also suggest potential sex-based differences in conduction outcomes. Female patients may have a higher risk of developing LBBB following TAVI, particularly when smaller prostheses are implanted or in the presence of pre-existing conduction system fragility. In parallel, GA has been associated with bradyarrhythmias and greater autonomic modulation during valve deployment, potentially increasing susceptibility to conduction disturbances compared to MAC. However, these relationships remain incompletely understood and are likely influenced by multiple interacting factors, including valve type, implantation depth, annular oversizing, and individual anatomic variation.

### 4.3. Study Limitations

We consider the study’s limitations below. The retrospective design limits the ability to capture additional variables, potentially resulting in unmeasured confounding, while the extended study period (2011–2023), required to achieve adequate power, may introduce measurement bias. Additionally, while all gradients were echocardiographically derived, our study did not standardize the measurement conditions (e.g., sedation level, heart rate, blood pressure, ventilation mode) across time points. This reflects the retrospective nature of the dataset, and we acknowledge that variation in these parameters may have influenced gradient interpretation. We are also precluded from examining longer follow-up (1–2 years), which would clarify whether early sex-based hemodynamic differences impact valve durability or clinical outcomes, as outcomes at these time points were not routinely collected between 2011 and 2023. Furthermore, our dataset did not include post-procedural ECG data, precluding us from evaluating the incidence of conduction disturbances such as atrioventricular block (AVB) or left bundle branch block (LBBB). This is a relevant omission. Future studies incorporating routine ECG follow-up would allow a more comprehensive assessment of potential sex- or anesthesia-related differences in conduction disturbances, which could further refine procedural planning and post-operative care.

In addition, the four sex and anesthesia type groups differed significantly in several baseline clinical characteristics, complicating direct comparisons. Our data come from a single center in East Tennessee, which may limit generalizability to other populations, introduce potential selection bias, and contribute to small numbers within each of the sex and anesthesia type groups. With that said, we note that the University of Tennessee Medical Center is a large regional academic hospital with a catchment area that encompasses all of East Tennessee as well as portions of Southeast Kentucky and Western North Carolina [26]. Importantly, the fact that our data are derived from a single center does not inherently make the study more vulnerable to missing data or unmeasured confounding than multicenter studies. The degree of missing data and unmeasured confounding in both single and multicenter studies is also influenced by the data collection process, the uniformity of follow-up on patient responses, and the overall study design (observational versus experimental, prospective versus retrospective) [27].

The exclusion of patients who crossed over from MAC to GA during TAVI may also reduce the applicability of our findings to this subgroup. In addition, this exclusion may bias the sample toward more stable patients, which could make our study estimates more conservative. Nonetheless, our study is the first to examine immediate and post-surgical anesthesia response at 30 days and six months by sex among patients undergoing TAVI, providing insights that may improve the quality of care for this growing patient population.

## 5. Conclusions

In this retrospective cohort study, we assessed how sex, anesthesia type, and valve size relate to early post-TAVI hemodynamic changes. Although unadjusted analyses initially suggested that female patients experienced greater increases in transvalvular gradients 24 h after TAVI, further investigation revealed that these differences were primarily attributable to valve size, which varied systematically across sex-anesthesia groups. After adjusting for valve size in multivariable models, no significant differences in gradient change remained between groups, indicating that valve size, rather than sex or anesthesia type, was the main driver of early hemodynamic variation. While all groups maintained normotensive blood pressures and exhibited no sex-based differences in vasopressor use within anesthesia categories, we acknowledge that brief peri-induction hypotensive episodes cannot be excluded. However, these events are unlikely to explain the observed gradient changes independently. Future prospective studies with larger, more balanced cohorts, particularly those including men with smaller valves, along with serial echocardiographic assessments and longer-term outcome data, are needed to further elucidate these relationships and their clinical implications. Our findings also motivate future investigation of how LV mass regression, functional status, and biomarkers could further shape understanding of the clinical impact of early gradient increases.

## Figures and Tables

**Table 1:** Baseline demographic and clinical characteristics of patients by sex and anesthesia type (*n* = 693).

Characteristic	Male		Female		*p*-Value
	General Anesthesia (*n* = 113)	Monitored Anesthesia Care (*n* = 280)	General Anesthesia (*n* = 78)	Monitored Anesthesia Care (*n* = 222)	
Age (Years)	77 [71–83]	76 [71–82]	80 [72–86]	77 [71–83]	0.097
Clinical History					
Diabetes Mellitus	61 (54.0%)	120 (42.9%)	38 (48.7%)	88 (39.6%)	0.07
Hypertension	108 (95.6%)	246 (87.9%)	71 (91.0%)	194 (87.4%)	0.06
Hyperlipidemia	104 (92.0%)	236 (84.3%)	65 (83.3%)	188 (84.7%)	0.15
Peripheral vascular disease	65 (57.5%)	58 (20.7%)	26 (33.3%)	35 (15.8%)	<0.001
Stroke/TIA	19 (16.8%)	23 (8.2%)	19 (24.4%)	35 (15.8%)	0.001
COPD	35 (31.0%)	44 (15.7%)	20 (25.6%)	32 (14.4%)	<0.001
Atrial fibrillation	37 (32.7%)	79 (28.2%)	33 (42.3%)	59 (26.6%)	0.06
Previous pacemaker	14 (12.4%)	36 (12.9%)	10 (12.8%)	21 (9.5%)	0.65
CKD (any stage)	58 (51.3%)	93 (33.2%)	57 (73.1%)	94 (42.3%)	<0.001
CKD stage 4 or ESRD	10 (8.8%)	14 (5.0%)	15 (19.2%)	20 (9.0%)	0.003
GFR	60 [43–78]	72 [57–87]	47 [34–60]	65 [46–82]	<0.001
Coronary artery disease	97 (85.8%)	176 (62.9%)	49 (62.8%)	102 (45.9%)	<0.001
Previous myocardial infarction	45 (39.8%)	37 (13.2%)	16 (20.5%)	13 (5.9%)	<0.001
Previous CABG	42 (37.2%)	51 (18.2%)	13 (16.7%)	15 (6.8%)	<0.001
Obstructive sleep apnea	32 (28.3%)	55 (19.6%)	11 (14.1%)	38 (17.1%)	0.06
BMI (kg/m^2^)	29 [26–35]	29 [25–33]	29 [25–36]	30 [25–36]	0.597
NYHA class 1–4 symptoms	3.0 [3.0–3.0]	3.0 [3.0–3.0]	3.0 [3.0–3.0]	3.0 [3.0–4.0]	0.002
CHA2DS2-VASc Score	5 [4–5]	4 [3–5]	6 [4–6]	5 [4–6]	<0.001

Abbreviations: BMI, body mass index; CABG, coronary artery bypass graft; CKD, chronic kidney disease; COPD, chronic obstructive pulmonary disease; ESRD, end-stage renal disease; GFR, glomerular filtration rate; NYHA, New York Heart Association; TIA, transient ischemic attack. Data is expressed as median [IQR, 25th–75th percentile] or proportion (percentages). *p* < 0.05 indicates the difference between the two groups is statistically significant.

**Table 2:** Hemodynamics, anesthesia, and echocardiographic characteristics of patients by sex and anesthesia type.

Characteristic	Male		Female		*p*-Value
	General Anesthesia (*n* = 113)	Monitored Anesthesia Care (*n* = 280)	General Anesthesia (*n* = 78)	Monitored Anesthesia Care (*n* = 222)	
Hemodynamics and Anesthesia					
SBP pre-TAVI (mmHg)	149.0 [125.0–163.0]	151.5 [130.0–170.0]	149.5 [130.0–171.0]	156.0 [39.0–180.0]	0.022
DBP pre-TAVI (mmHg)	67.0 [57.0–75.0]	72.0 [62.0–81.0]	65.0 [56.0–74.0]	71.0 [63.0–82.0]	<0.001
SBP immediate post-TAVI (mmHg)	127.0 [114.0–143.0]	123.0 [111.0–140.0]	129.5 [116.0–150.0]	124.0 [111.0–137.0]	0.070
DBP immediate post-TAVI (mmHg)	53.0 [47.0–65.0]	62.0 [52.0–69.0]	53.5 [46.0–63.0]	56.0 [50.0–63.0]	<0.001
SBP 24 h post-TAVI (mmHg)	125.0 [117.0–138.0]	131.0 [118.0–142.0]	127.5 [118.0–146.0]	130.0 [117.0–140.0]	0.232
DBP 24 h post-TAVI (mmHg)	57.0 [50.0–63.0]	63.0 [58.0–71.5]	57.5 [49.0–65.0]	60.0 [54.0–67.0]	<0.001
Total fentanyl dose (mcg)	250.0 [150.0–500.0]	0.0 [0.0–62.5]	250.0 [150.0–600.0]	0.1 [0.0–100.0]	<0.001
Total versed dose (mg)	2.0 [0.0–3.0]	0.0 [0.0–1.0]	2.0 [0.0–3.0]	0.0 [0.0–1.0]	<0.001
Propofol bolus (mg)	0.0 [0.0–50.0]	0.0 [0.0–0.0]	0 [0.0–50.0]	0.0 [0.0–0.0]	<0.001
Propofol infusion (mg)	0.0 [0.0–77.6]	0.0 [0.0–0.0]	0.0 [0.0–67.9]	0.0 [0.0–0.0]	<0.001
Total propofol (mg)	45.4 [0.0–104.0]	0,0 [0.0–0.0]	50.0 [0.0–100.0]	0 [0.0–0.0]	<0.001
Dexmedetomidine bolus (mcg)	0.0 [0.0–0.0]	75.0 [35.0–100.0]	0.0 [0.0–0.0]	60.0 [31.0–90.0]	<0.001
Dexmedetomidine infusion (mcg)	0.0 [0.0–0.0]	55.6 [32.7–92.8]	0.0 [0.0–0.0]	49.3 [28.7–73.4]	<0.001
Total Dexmedetomidine used (mcg),	0.0 [0.0–0.0]	124.1 [95.1–168.2]	0.0 [0.0–0.0]	109.5 [72.0–148.5]	<0.001
Use of inhaled anesthetic	112 (99.1%)	0 (0.0%)	78 (100.0%)	0 (0.0%)	<0.001
Need for vasopressors	92 (81.4%)	93 (33.2%)	59 (75.6%)	89 (40.1%)	<0.001
IV fluids during TAVI (ml)	750.0 [400.0–1000.0]	500.0 [233.0–650.0]	575.0 [300.0–800.0]	500.0 [250.0–700.0]	<0.001
24 h fluid balance	856.0 [100.0–1931.5]	434.5 [−221.8- 922.0]	704.5 [10.9.0–1503.5]	541.0 [68.0–1028.0]	<0.001
Pre-TAVI weight	88.9 [77.0–103.4]	89.0 [78.3–102.8]	74.6 [64.0–92.3]	74.8 [63.0–92.5]	<0.001
Post TAVI weight	90.7 [76.8–104.8]	88.4 [77.1–101.5]	76.6 [64.1–93.8]	74.9 [63.5–93.0]	<0.001
Echocardiographic					
Pre-TAVI ECHO mean gradient (mmHg)	41.0 [30.0–46.0]	37.6 [29.3–44.6]	41.6 [30.7–48.8]	40.1 [30.9–46.0]	0.073
Pre-TAVI ECHO AVA (cm^2^)	0.8 [0.7–0.9]	0.8 [0.7–0.9]	0.8 [0.6–0.9]	0.8 [0.6–0.9]	0.040
Pre-TAVI EF (%)	55.0 [45.0–59.8]	60 [53–64.5]	55.9 [55.0–62.0]	60.0 [56.0–65.0]	<0.001
Pre-TAVI AR severity	21 (18.6%)	37 (13.2%)	11 (14.1%)	33 (14.9%)	0.61
Post-TAVI EF	55 [45.4–60.0]	60.0 [55.0–65.0]	60.0 [55.0–63.0]	63.4 [58.6–67.0]	<0.001
Post-TAVI AVA	1.7 [1.5–2.0]	1.7 [1.4–2.1]	1.8 [1.4–2.1]	1.5 [1.3–1.9]	<0.001
Immediate TAVI mean gradient	4.1 [3.0–6.0]	4.0 [3.0–6.0]	4.1 [3.0–5.6]	5.0 [4.0–7.0]	<0.001
24 h TAVI mean gradient	9 [6.6–12.0]	9.7 [6.9–13.1]	11.0 [8.6–15.0]	12.0 [9.1–16.7]	<0.001
Mean gradient delta	4.4 [2.4–7.2]	5.0 [2.9–8.0]	6.4 [4.1–9.8]	7.1 [4.0–10.8]	<0.001
Immediate TAVI peak gradient	9 [7.0–12.0]	8.0 [6.0–11.9]	9.0 [6.1–12.0]	10.7 [7.8–14.8]	<0.001
24 h TAVI peak gradient	18.2 [14.0–24.5]	19.0 [13.3–25.0]	22.4 [7.4–30.0]	24.1 [17.5–32.0]	<0.001
Max gradient delta	9.7 [3.9–15.0]	10.4 [5.5–18.0]	13.4 [8.2–18.2]	15.0 [8.0–22.8]	<0.001
30-day TAVI mean gradient	10.0 [6.5–11.4]	10.0 [7.1–13.1]	10.2 [7.4–14.9]	12.4 [9.0–16.5]	<0.001
30-day TAVI peak gradient	19.8 [14.1–24.0]	19.6 [15.0–27.0]	20.9 [16.1–29.9]	25.0 [17.1–31.9]	<0.001
30-day EF	55.0 [47.7–57.0]	58.0 [55.0–63.0]	55.0 [53.5–60.0]	60.0 [56.0–65.0]	<0.001
Presence of paravalvular leak	22 (19.5%)	16 (5.7%)	18 (23.1%)	9 (4.1%)	<0.001
Severity of paravalvular leak (trace–mild)	20 (17.7%)	14 (5.0%)	16 (20.5%)	11 (5.0%)	<0.001
Severity of paravalvular leak (moderate–severe)	1 (0.9%)	1 (0.4%)	0 (0.0%)	0 (0.0%)	0.45

Abbreviations: AR, aortic regurgitation; AVA, aortic valve area; DBP, diastolic blood pressure; EF, ejection fraction; IV, intravenous; SBP, systolic blood pressure; TAVI, transcatheter aortic valve implantation. Data is expressed as median [IQR, 25th–75th percentile] or proportion (percentages). *p* < 0.05 indicates the difference between the two groups is statistically significant.

**Table 3:** Length of stay and 6-month outcomes post-TAVI by sex and anesthesia type.

Outcome	Male		Female		*p*-Value
	General Anesthesia (*n* = 113)	Monitored Anesthesia Care (*n* = 280)	General Anesthesia (*n* = 78)	Monitored Anesthesia Care (*n* = 222)	
Post-TAVI length of stay (days)	2 [2–5]	1 [1–2]	3 [2–4]	2 [1–2]	<0.001
MI post-TAVI, 6 months	1 (0.9%)	1 (0.4%)	0 (0.0%)	0 (0.0%)	0.45
Stroke/TIA post-TAVI, 6 months	5 (7.7%)	5 (1.8%)	6 (7.7%)	7 (3.2%)	0.10
All cause death post-TAVI 6 months	6 (5.3%)	9 (3.2%)	4 (5.1%)	8 (3.6%)	0.74
CV death post-TAVI, 6 months	2 (0.7%)	5 (4.4%)	2 (2.6%)	4 (1.8%)	0.12

Abbreviations: MI, myocardial infarction; CV, cardiovascular; TAVI, transcatheter aortic valve implantation. Data is expressed as median [IQR, 25th–75th percentile] or proportion (percentages). *p* < 0.05 indicates the difference between the two groups is statistically significant.

**Table 4:** Pairwise comparisons of continuous characteristics/outcomes by sex and anesthesia type.

Characteristic/Outcome	MAC-Male and GA-Male	MAC-Male and MAC-Female	MAC-Male and GA-Female	GA-Male and MAC-Female	GA-Male and GA-Female	MAC-Female and GA-Female
Clinical History						
NYHA class I–IV symptoms		✓	✓			
CHA2DS2-VASc Score	✓	✓	✓		✓	
Hemodynamics and Anesthesia						
SBP pre-TAVI (mmHg)				✓		
DBP pre-TAVI (mmHg)	✓		✓	✓		✓
DBP immediate post-TAVI (mmHg)	✓	✓	✓			
DBP 24 h post-TAVI (mmHg)	✓	✓	✓	✓		
Total fentanyl dose (mcg)	✓		✓	✓		✓
Total versed dose (mg)	✓		✓	✓		✓
Propofol bolus (mg)	✓		✓	✓		✓
Propofol infusion (mg)	✓		✓	✓		✓
Total propofol (mg)	✓		✓	✓		✓
Dexmedetomidine bolus (mcg)	✓		✓	✓		✓
Dexmedetomidine infusion (mcg)	✓		✓	✓		✓
Total Dexmedetomidine used (mcg)	✓		✓	✓		✓
IV fluids during TAVI (ml)	✓			✓		
24 h fluid balance	✓		✓	✓		
Pre-TAVI weight		✓	✓	✓	✓	
Post TAVI weight		✓	✓	✓	✓	
Echocardiographic						
Pre-TAVR ECHO AVA (cm^2^)		✓				
Pre-TAVR EF (%)	✓	✓		✓	✓	✓
Post-TAVR EF	✓	✓		✓	✓	✓
Post-TAVR AVA		✓		✓		✓
Immediate TAVI mean gradient		✓		✓		✓
24 h TAVI mean gradient		✓	✓	✓	✓	
Mean gradient delta		✓		✓	✓	
Immediate TAVI peak gradient		✓				
24 h TAVI peak gradient		✓	✓	✓	✓	
Peak gradient delta		✓		✓	✓	
30-day TAVI mean gradient		✓		✓		
30-day TAVI peak gradient				✓		✓
30-day EF	✓	✓		✓		✓
Outcomes						
Post-TAVI length of stay (days)	✓	✓	✓	✓		✓

Abbreviations: AVA, aortic valve area; DBP, diastolic blood pressure; EF, ejection fraction; IV, intravenous; NYHA, New York Heart Association; SBP, systolic blood pressure; TAVI, transcatheter aortic valve implantation. Data are expressed as median [IQR, 25th–75th percentile] or proportion (percentages). Shaded box and checkmark stand for *p* < 0.05, indicating that the difference between the two sex and anesthesia groups is statistically significant.

## Data Availability

The data presented in this study are available on request from the corresponding author.

## Supplementary Materials

The following supporting information can be downloaded at: https://www.mdpi.com/article/10.3390/jcm14196693/s1. Figure S1. A forest plot illustrating the multivariable regression analysis of predictors of mean gradient delta; Table S1. Pairwise comparison of mean gradient delta by valve size (categorical variable) *; Table S2. Pairwise Comparisons of Valve Size (ordinal variable) by Sex and Anesthesia type **; Table S3. Negative binomial regression modeling mean gradient delta outcome with group status, valve size and other anesthesia- and sex-related covariates.

## Abbreviations

The following abbreviations are used in this manuscript:

AKI	Acute kidney injury
AR	Aortic regurgitation
AVA	Aortic valve area
BMI	Body mass index
CABG	Coronary artery bypass graft
CKD	Chronic kidney disease
COPD	Chronic obstructive pulmonary disease
CV	Cardiovascular
DBP	Diastolic blood pressure
EF	Ejection fraction
ESRD	End-stage renal disease
GA	General anesthesia
GFR	Glomerular filtration rate
IQR	and interquartile ranges
IV	Intravenous
MDN	Medians
MI	Myocardial infarction
NYHA	New York Heart Association
SBP	Systolic blood pressure
SVR	Systemic vascular resistance
TAVI	Transcatheter aortic valve implantation
TIA	Transient ischemic attack
TV	Transvalvular
