# Supplementary material for: Sex-Based Differences in Hemodynamic Response to Anesthesia Type During TAVI and Early Transvalvular Gradient Changes
Source: J Clin Med. 2025 Sep 23;14(19):6693. doi: 10.3390/jcm14196693 (PMC12525419; doi:10.3390/jcm14196693)
Supplement: Supplementary file 1 [file jcm-14-06693-s001.zip › jcm-3820506-supplementary.pdf]

Figure S1. A forest plot illustrating the multivariable regression analysis of predictors of mean gradient delta

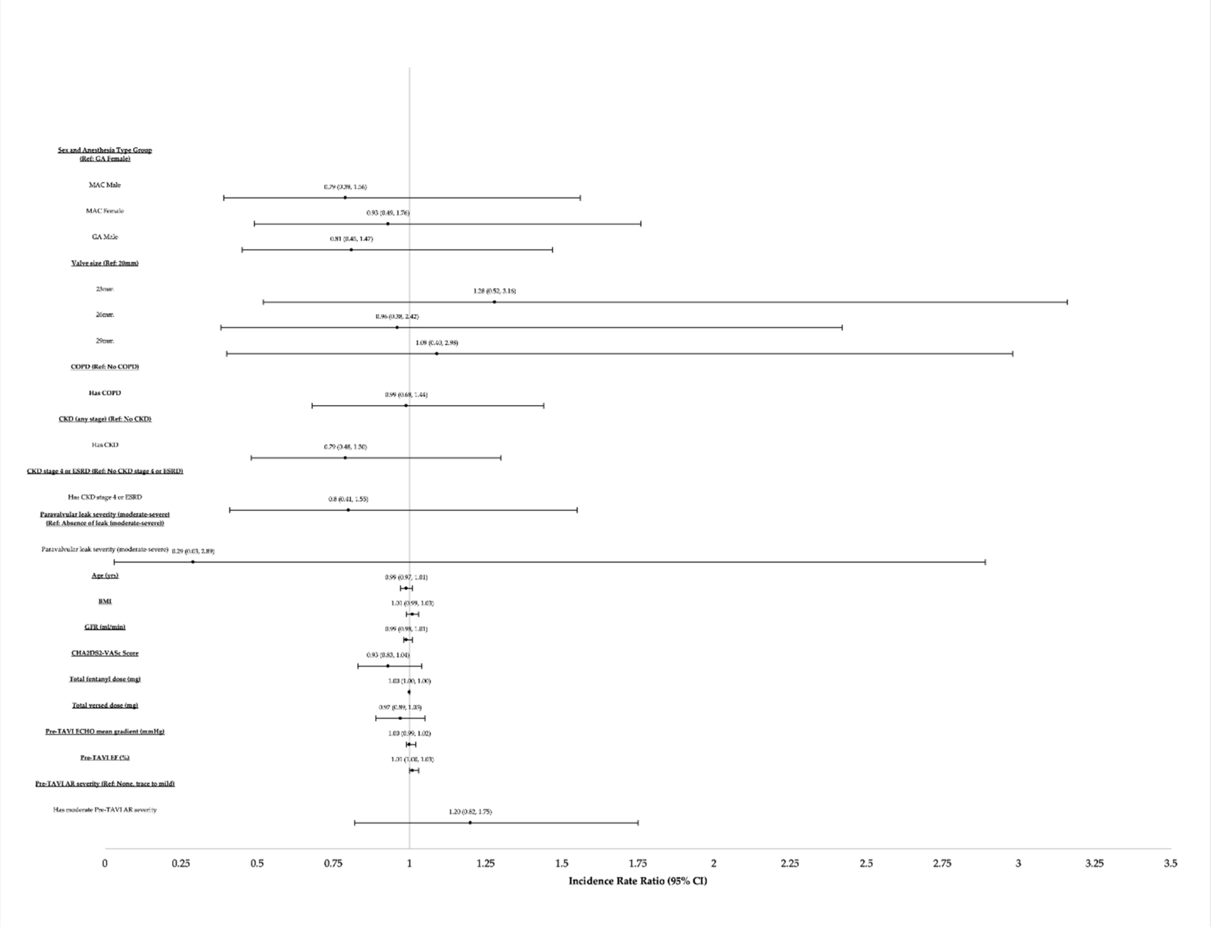

Table S1. Pairwise comparison of mean gradient delta by valve size (categorical variable)*

Characteristic/Outcome	20mm and 23mm	20mm and 26mm	20mm and 29mm	23mm and 26mm	23mm and 29mm	26mm and 29mm
Mean Gradient delta		✓	✓	✓	✓	

Table S2. Pairwise Comparisons of Valve Size (ordinal variable) by Sex and Anesthesia type**

Characteristic/Outcome	MAC-Male and GA-Male	MAC-Male and MAC-Female	MAC-Male and GA-Female	GA-Male and MAC-Female	GA-Male and GA-Female	MAC-Female and GA-Female
**Valve Size**	✓	✓	✓	✓	✓	

Table S3. Negative binomial regression modeling mean gradient delta outcome with group status, valve size and other anesthesia- and sex-related covariates.

Parameter	Incidence Rate Ratio (95% Confidence Interval)	P-value
**Sex and Anesthesia Type Group (Ref: GA Female)**		
**MAC Male**	0.79 (0.39, 1.56)	0.492
**MAC Female**	0.93 (0.49, 1.76)	0.828
**GA Male**	0.81 (0.45, 1.47)	0.484
**Valve size (Ref: 20mm)**		
**23mm**	1.28 (0.52, 3.16)	0.597
**26mm**	0.96 (0.38, 2.42)	0.934
**29mm**	1.09 (0.40, 2.98)	0.864
**COPD (Ref: No COPD)**		
**Has COPD**	0.99 (0.68, 1.44)	0.946
**CKD (any stage) (Ref: No CKD)**		
**Has CKD**	0.79 (0.48, 1.30)	0.360
**CKD stage 4 or ESRD (Ref: No CKD stage 4 or ESRD)**		
**Has CKD stage 4 or ESRD**	0.8 (0.41, 1.55)	0.507
**Severity of paravalvular leak (moderate–severe) (Ref: Absence of Severity of perivalvular leak (moderate-severe))**		
**Has severity of paravalvular leak (moderate–severe)**	0.29 (0.03, 2.89)	0.289
**Age (yrs)**	0.99 (0.97, 1.01)	0.421
**BMI**	1.01 (0.99, 1.03)	0.462
**GFR (ml/min)**	0.99 (0.98, 1.01)	0.334
**CHA2DS2-VASc Score**	0.93 (0.83, 1.04)	0.184
**Total fentanyl dose (mg)**	1.00 (1.00, 1.00)	0.489
**Total versed dose (mg)**	0.97 (0.89, 1.05)	0.472
**Pre-TAVI ECHO mean gradient (mmHg)**	1.00 (0.99, 1.02)	0.572
**Pre-TAVI EF (%)**	1.01 (1.00, 1.03)	0.066
**Pre-TAVI AR severity (Ref: None, trace to mild)**		
**Has moderate Pre-TAVI AR severity**	1.20 (0.82, 1.75)	0.355

* Shaded box and checkmark stands for $p < 0.05$ indicating the difference between the two valve size groups is statistically significant; ** Shaded box and checkmark stands for $p < 0.05$ indicating the difference between the two sex and anaesthesia groups is statistically significant.
